# Looking Back and Going Forward: Roles of Varenicline and Electronic Cigarettes in Smoking Cessation

**DOI:** 10.7759/cureus.16824

**Published:** 2021-08-02

**Authors:** Ebenezer O Oloyede, Olatunde Ola, Victor O Kolade, Justin Tevie

**Affiliations:** 1 Internal Medicine, Anne Arundel Medical Center, Annapolis, USA; 2 Hospital Medicine, Mayo Clinic Health System, La Crosse, USA; 3 Center for Clinical and Translational Science, Mayo Clinic Graduate School of Biomedical Sciences, Rochester, USA; 4 Medicine, Guthrie Clinic/Robert Packer Hospital, Sayre, USA; 5 Health Economics, Missouri Department of Mental Health, Jefferson City, USA

**Keywords:** smoking cessation, e-cigarette, varenicline, public health, cardiovascular disease, nicotine addiction, electronic nicotine delivery systems (ends), e-cigarette or vaping use-associated lung injury (evali), health policy, pulmonary disease

## Abstract

Tobacco use is the single largest preventable cause of death in the United States (US). The national goal of reducing the prevalence of adult cigarette smoking to 12% was retained for 20 years due to non-attainment. Meanwhile, varenicline and electronic cigarettes (ECs) became available in the US in 2006 and 2007, respectively, and have been used by many smokers wanting to quit. The purpose of this review is to compare varenicline and ECs in terms of efficacy for smoking cessation after over a decade of widespread use in the US.

Data collection for systematic review and qualitative synthesis by a PubMed search using Preferred Reporting Items for Systematic Reviews and Meta-Analyses (PRISMA) guidelinesand the Oxford Quality Scale, respectively, was performed in June 2018 and updated in June 2020. Articles were eligible if published in English as original research in the form of a randomized clinical trial (RCT), a systematic review and meta-analysis, a systematic review, or a cross-sectional study.

Eighteen studies were included: nine RCTs, four cross-sectional studies, two meta-analyses, one systematic review, one systematic review and meta-analysis, and one cohort study. No head-to-head RCT compared varenicline to ECs. In four RCTs, varenicline was more effective than placebo for smoking cessation. In two RCTs, ECs were more effective than placebo but a meta-analysis of 20 studies reported a statistically significant decrease in the odds of quitting smoking using ECs as compared to placebo.

To conclude, varenicline and ECs have data suggesting efficacy for smoking cessation; however, unlike varenicline, ECs were not effective in all studies.

## Introduction and background

Smoking cost the United States (US) $169 billion in annual healthcare expenditures by 2010 [1] and was the leading cause of death in 2000 [2]. The decline in cigarette smoking prevalence among US adults from 21% in 2005 to 13.7% in 2018 [3] falls short of national Healthy People objectives, and the quest to end the tobacco use epidemic espoused by the 2020 report of the Surgeon General on smoking cessation [4]; therefore, reduction in smoking prevalence rates remains a key public health goal [5-6]. Several clinician-level and community-level interventions remain in effect: bans on smoking in public places, high cigarette taxes, restriction on tobacco sales to minors, and a Public Health Service (PHS) guideline that recommends tobacco dependence be considered a chronic condition that requires repeated intervention [5,7]. Hence, all smokers should receive practical cessation counseling combined with appropriate pharmacotherapy; this combination produces quit rates five times higher than quitting without assistance [8].

Unfortunately, population-based research shows that most smokers who report making quit attempts do so without the benefit of recommended counseling and pharmacotherapy [5,9]. Guideline-compliant counseling includes the 5As - ask, advise, assess, assist, and arrange follow-up - and implies a discussion of best/evidence-based methods of quitting tobacco use; varenicline, bupropion, and nicotine replacement therapy (NRT) are the first-line medications recommended by the PHS.

Electronic cigarettes or e-cigarettes (ECs) are battery-powered devices that consist of a tank holding a nicotine-containing liquid solution (e-liquid or e-juice), a heating element, and a mouthpiece through which heated liquid solution vaporizes and is inhaled by the user [10]. The liquid solution usually contains propylene glycol/glycerin and other chemicals, which may include nickel, cadmium, lead, methylbenzaldehyde, and flavors - items not found in cigarette smoke [11]. E-cigarettes became commercially available to United States consumers in 2007 [10] and gained popularity over the following decade; forms of ECs have changed with time, and nicotine contents of included solutions vary [11]. ECs were initially marketed by online vendors as smoking cessation aids. Although they are not included as first- or second-line cessation aids by the PHS guideline [5], e-cigarettes have become quite popular among smokers. By 2013-2014, major tobacco companies had jumped into EC distribution, looking for a new source of growth [11-12]; in 2018, 3.2% of US adults were current users of e-cigarettes [3]. Electronic cigarettes were initially minimally regulated; they were not labeled as a tobacco product until 2016 when the US Food and Drug Administration (FDA) issued a new tobacco rule [13].

This rule extends its regulatory jurisdiction to all tobacco products, including electronic cigarettes, all cigars (including premium ones), hookah (also called waterpipe tobacco), pipe tobacco, nicotine gels, and 'dissolvables' that did not previously fall under the Food and Drug Administration’s (FDA’s) jurisdiction as part of its efforts to protect the American public from tobacco-related diseases and death. Some of its provisions include evaluation of important factors, such as ingredients, product design, and health risks, as well as the products’ appeal to youth and non-users; mandatory health warnings on roll-your-own tobacco, cigarette tobacco, and certain newly regulated tobacco products; bans on all free samples; tobacco products not on the market by February 15, 2007, require pre-authorization from the FDA and must demonstrate that such products meet stipulated public health standards; restriction of the sale of newly regulated products to minors (<18-year-old) directly or through vending machines, as well as mandatory age verification with a government-issued photo identification card.

Prior to this rule, there was no federal law to stop retailers from selling e-cigarettes to youth under age 18; despite the rule, the current use of ECs by middle and high school students increased two- to three-fold from 2017 to 2019 [14]. Subsequently, a ban on most flavored e-cigarettes was announced by the FDA in January 2020 [15]. In addition, the U.S. Congress has recently raised the minimum age for the purchase of all tobacco products, including e-cigarettes, to 21 years [16].

When varenicline was approved by the FDA in 2006, it boasted higher quit rates than all previously approved cessation pharmacotherapy [17-18]. Varenicline is a prescription-only drug in the US, while e-cigarettes have been on the open market (Table 1). The impact of these two contemporary additions to the tobacco control scene on smoking prevalence is reviewed to predict their potential effects on cessation rates in the years ahead [5,10-11,13-14,19-28].

**Table 1:** Comparing varenicline with E-cigarettes: pros and cons

Product	Pros	Cons
E-cigarettes	1. Requires no prescription (OTC); dose is controlled by the user	1. Not endorsed by clinical guidelines or specialty position statements [13, 19, 20, 21]
	2. Some demonstrated benefits for smoking cessation [22]	2. Cost (not covered by health insurance)
	3. Widely available [14]	3. Heterogenous components [10-11]
	4. Presumably less toxic than conventional cigarettes [23]	4. Inconclusive evidence on safety and efficacy[4,23]
	5. Attractive to youth and young adults [14]	5. Neither health professionals nor users are fully aware of long-term side effects [23-24]
	6. Usage has increased year-over-year [14]	6. Not studied in clinical trials prior to availability to smokers
Varenicline	1. Most effective smoking cessation pharmacotherapy [25]	1. Prescription-only medication [5]
	2. FDA-approved[5]	2. Cost (not covered 100% by insurance) [26]
	3. Recommended in clinical guidelines at a specific dose, 2 mg/d [5]	3. Pharmacology: Slower onset of action, so not ideal for in-patient setting - yet showing long-term efficiency [27-28]
	4. Black box warning on neuropsychiatric side effects removed by FDA based on post-marketing data [25]	4. Documented side-effects (e.g. headaches, mood changes, seizures) are known to users [28]

## Review

Methods

**Figure 1:**
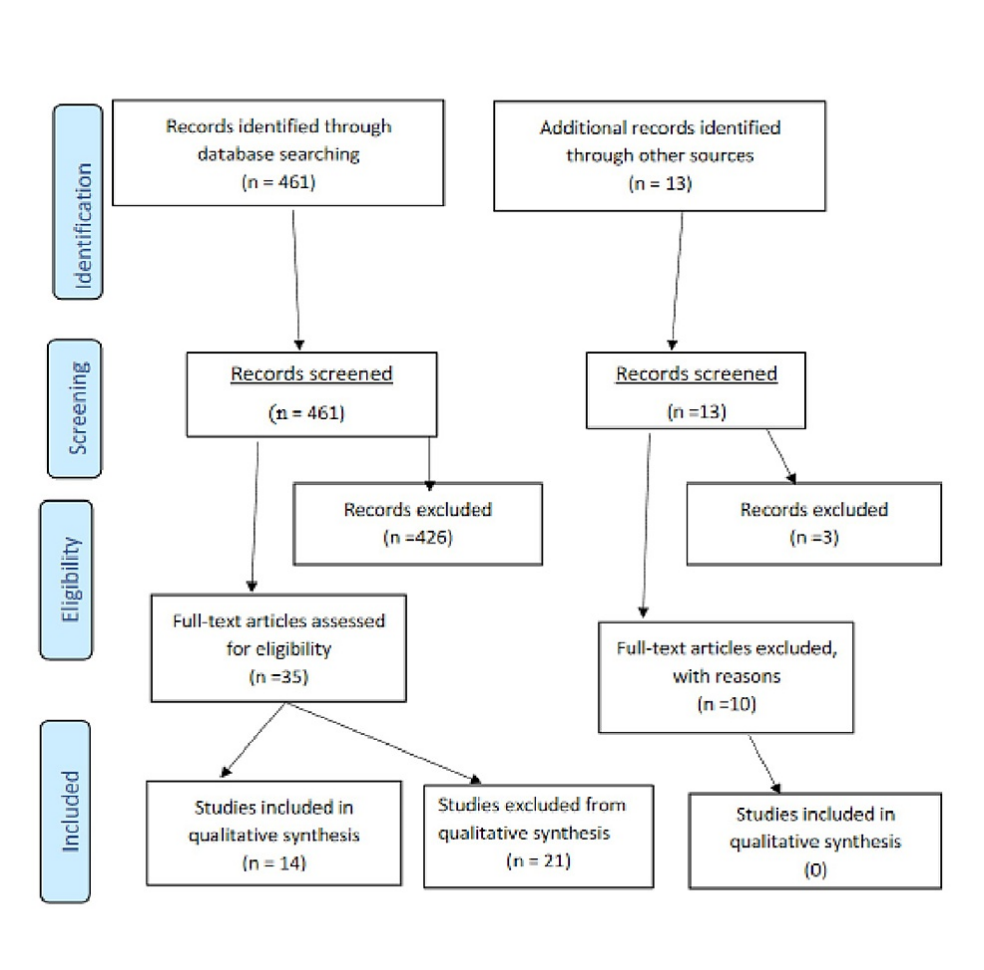
PRISMA article selection flow diagram PRISMA: Preferred Reporting Items for Systematic Reviews and Meta-Analyses Source: [29]

We conducted a systematic review following a literature search of relevant articles in June 2018 using the PubMed Advanced Search Builder with the following MeSH search terms: “((Varenicline) AND E-cigarettes) AND Smoking Cessation”, “(E-cigarettes) AND Regulation”, and ((E-cigarettes) AND Efficacy) AND side effects. Citations from relevant articles were further reviewed to identify manuscripts that may have been missed in the initial literature search. Using Preferred Reporting Items for Systematic Reviews and Meta-Analyses (PRISMA) guidelines (Figure 1) [29], we identified articles that met the following eligibility criteria for systematic review and qualitative synthesis: 1. English Language; 2. Original Research; 3. Randomized Clinical Trials; 4. Systematic Review and Meta-Analysis; 5. Systematic Review; 6. Cross-Sectional Online Survey. We also summarized the comparison between varenicline and e-cigarettes using information from available studies done on each product (Tables 1-3). We performed an updated data abstraction in June 2020. Each of the nine (9) randomized control trials (RCTs) included in our review was scored for quality using the Oxford Quality Scale created by Jadad et al. [30]. The scale comprised five questions, with a maximum attainable score of five (5) for each randomized trial. Each question either added (‘Y’) or subtracted a point (‘N’) from a study: (a) Is the study randomized? If “yes” one point given; otherwise one point deducted; (b) Is the randomization procedure reported and appropriate? if “yes”, one point given, if “no” one point deducted; (c) Is the study double blind? If “yes” one point given; otherwise one point deducted; (d) Is the blinding procedure appropriate and adequate? If “yes” one point given. If “no” one point deducted; and (e) Are withdrawals and dropouts described? If “yes” one point given, otherwise no point given if withdrawals are not mentioned.

**Table 2:** Summary of varenicline studies

	Study	Study Design	Sample Size (N)/ Odds Ratio (OR) (or Relative Risk, RR)	Comment
1.	Jorenby et al, 2006 [17]	Randomized controlled trial	N = 1027. Continuous smoking abstinence rates in the last 4 of 12 weeks of treatment were significantly better with varenicline compared to placebo (OR: 3.85; 95% CI, 2.69-5.50) and Bupropion (OR, 1.90; 95% CI, 1.38-2.62).	Varenicline was a safe and more effective smoking cessation pharmacotherapy than bupropion and placebo.
2.	Gonzales et al, 2006 [18]	Randomized controlled trial	N = 1025. The 7-day continuous quit rates were significantly greater for Varenicline compared to placebo at weeks 12, 24, and 52 (P <0.001). Also, significantly higher for varenicline compared to bupropion at weeks 12 and 24 (P <0.01).	Varenicline was significantly more efficacious than bupropion and placebo for smoking cessation.
3.	Anthenelli et al, 2016 [25]	Randomized, placebo-controlled clinical trial	N = 8144. Varenicline showed greater abstinence rates than those on placebo (OR 3·61, 95% confidence interval [CI] 3·07 - 4·24), nicotine patch (OR 1·68, CI 1·46 -1·93), and bupropion (OR 1·75, CI 1·52 to 2·01).	Varenicline was not associated with increased neuropsychiatric events and demonstrated greater effectiveness than bupropion, nicotine patch, and placebo.
4.	Ebbert et al, 2015 [31]	Randomized clinical trial	N = 1510. A significant percentage of varenicline-treated participants reduced smoking by >75% compared to placebo after 8 weeks (RR = 1.8; 95% CI 1.4-2.2). Varenicline group also had higher smoking abstinence rates versus placebo after 6 months to 1 year (RR 2.7; 95% CI 2.1- 3.5).	Varenicline increased smoking reduction and smoking abstinence rates.
5.	Chang et al, 2015 [32]	Meta-analysis	Three randomized controlled trials with 904 participants. Varenicline with nicotine patch was associated with a significant increase in smoking cessation compared to varenicline plus placebo (OR = 1.62, 95 % CI 1.18 - 2.23).	Combination treatment with varenicline and nicotine is more effective than varenicline alone.
6.	Cahill et al, 2013 [33]	Meta-analysis	This covered 267 studies, involving 101,804 study subjects. Varenicline increased the odds of smoking cessation compared to NRT (OR 1.57; 95% CI 1.29 to 1.91), and bupropion (OR 1.59; 95% CI 1.29 to 1.96).	Varenicline was superior to single forms of NRT (OR 1.57) and to bupropion (OR 1.59).

**Table 3:** Summary of E-cigarette studies N = sample size, ECs = electronic cigarettes, OR = odds ratio, RCTs = randomized controlled trials, RR = relative risk, AOR = adjusted odds ratio

	Study	Study Design	Sample Size/ Odds Ratio	Comment
1.	Bullen et al, 2013 [22]	Randomized controlled trial	N = 657. At 6 months, smoking abstinence was highest with nicotine electronic cigarettes (7·3%); compared to nicotine patches (5·8%), and placebo e-cigarettes (4·1%). This difference was not statistically significant.	Nicotine electronic cigarettes were modestly effective at achieving smoking abstinence.
2.	Tseng et al, 2016 [37]	Randomized controlled trial	N = 99. E-cigarette subjects reduced smoking by 50% or more after 3 weeks of treatment (p<0.01). This effect was seen with nicotine ECs and placebo ECs. The quit rate at 3 weeks was 3%.	The use of ECs was preceded by telephone counseling designed to review current smoking patterns and offer change strategies.
3.	Adriaens et al, 2014 [38]	Randomized controlled trial	N = 48. A quit rate of 34% was noted among the two e-cigarette groups (n = 32) after 2 months compared to 0% in the control group (p < 0.01).	Second-generation ECs were effective in reducing nicotine dependence and inducing abstinence.
4.	Caponnetto et al, 2013 [39]	Randomized controlled trial	N = 300. Among subjects who used ECs for 12 weeks, 11% in the nicotine EC groups (n =200) and 4% in the placebo EC group (n=100) achieved smoking cessation at 1 year (p = 0.04).	26.9% of persons deemed to have quit smoking still used ECs at the end of the 1-year study.
5.	Siegel et al, 2011 [34]	Cross-sectional online survey	N = 216. Six months after the first purchase of Blu ECs, 31% of respondents were not smoking – though 57% of these were using ECs and 9% were using tobacco-free nicotine products.	Smoking abstinence rates increased with higher EC use; 70% of respondents who used ECs over 20 times a day were not smoking at 6 months.
6.	Kalkhoran et al, 2016 [35]	Systematic review and meta-analysis of 20 studies	Twenty studies were included in the meta-analysis. There was a 28% reduced odds of quitting smoking cigarettes among those who used electronic cigarettes compared to those who did not. (OR = 0·72, 95% CI 0·57–0·91).	The odds of smoking cessation were lower among EC users compared with those who did not use ECs.
7.	Rutten et al, 2015 [36]	Cross-sectional online survey	N = 2,254. Smokers who used ECs to try to quit smoking were more likely to report smoking fewer cigarettes since they started ECs (OR = 2.25; CI: 1.25–4.05).	No cessation outcome data was provided.
8.	McRobbie et al, 2014 [40]	Systematic review	Eleven cohorts and two RCTs were reviewed, including the two RCTs listed above [22,39]. Data pooled from the 2 RCTs (n = 662) showed that use of nicotine ECs was associated with higher abstinence rates than placebo ECs (RR 2.29, 95% CI 1.05-4.96). Pooled data from the same RCTs (n = 612) also showed that use of nicotine ECs was associated with a higher likelihood of a reduction in cigarette smoking by at least half compared to placebo ECs (RR 1.31, 95% CI 1.02-1.68).	Both RCTs included were found to be underpowered. All the cohort studies were deemed to have a high risk of bias.
9.	Brose et al, 2019 [41]	Longitudinal web-based survey	N = 374. former smokers followed for 15 months to assess characteristics affecting relapse. Survey of smokers and EC users. Overall, 39.6% relapsed. Compared with never use of ECs (35.9% relapse), neither past/ever use of ECs (45.9% relapse, OR =1.13; 95% CI: 0.61-2.07) nor daily use of ECs (34.5% relapse, OR=1.07; 95% CI: 0.61-1.89) were statistically different. Among EC users, the use of tank models was associated with higher odds of relapse than the use of modular devices (adjusted OR=3.63; 95% CI: 1.33-9.95)	Among ex-smokers who used ECs during the 15-month period, non-daily use was associated with higher odds of relapse than daily use.
10.	Levy et al, 2018 [42]	Cross-sectional study	N = 23633. Having made a quit attempt was more likely among smokers using e-cigarettes than non-users. Among those who made a quit attempt using e-cigarette: Ever used e-cigarettes (OR=2.31; CI: 2.15-2.48) EC use for 20+ days in last 30 days (OR=4.90; CI: 4.09-5.85) Significant differences were observed in quit success between EC use at different frequencies: never use (16.9%), ever but not current use (13.9 %), and for 1–4 days (5.2 %), 5–9 days (4.6 %), 10–14 days (6.6 %), 15–19 days (11.2 %), 20–24 days (17.1 %), and at least 25 days in the last month (32.6%). The adjusted OR of quit success was 59% higher with 5+ days of EC use and 181% higher with 20+ days of EC use. In linear form, the adjusted OR had a value of 1.10, indicating the odds of quit success increase by 10% with each additional day of use in the preceding 30 days.	A negative relationship was detected between quit success and e-cigarette ever use, but a positive relationship was observed with current e-cigarette use measures.
11.	McNeill et al, 2019 [43]	Cross-sectional survey	N=1070 former smokers. Current daily EC users were more likely than non-EC users to report: 1) having smoked within five minutes of waking (34.3% vs. 15.9%, AOR=3.74 (95% CI: 1.99-7.03), chi-square =16.92, p<0.001); having smoked >10 cigarettes/day (74.4% vs. 47.2%, AOR=4.39 (95% CI: 2.22-8.68), chi-square =18.18, p<0.001); 2) perceiving themselves to be still very addicted to smoking (41.3% vs. 26.2%, AOR=2.89 (95% CI: 1.58-5.30), chi-square =11.87, p<0.001) and 3) feeling extremely confident about staying quit (62.1% vs. 36.6%, AOR=3.22 (95% CI: 1.86-5.59), chi-square =17.36, p<0.001). EC users were not more likely to report any urges to smoke in the 24 hours preceding the survey than non-EC users (27.7% vs. 38.8%, AOR=0.86 (95% CI: 0.44-1.65), chi-square=0.21, p=0.643).	Compared to former smokers and current non-EC users, former smokers who currently use nicotine ECs daily reported higher levels of cigarette smoking dependence pre- and post-cessation, as well as greater confidence in staying quit and similar strength of urges to smoke (current users of non-nicotine ECs were excluded). 24.8% (n=166) of the non-EC users had used ECs at least weekly previously.
12.	Hajek et al, 2019 [44]	Randomized controlled trial	N = 886. current smokers 18% in the EC group had biochemically confirmed abstinence at 52 weeks, compared to 9.9% in the nicotine replacement group (RR 1.83; 95% CI 1.30-2.58).	75% of the study sample had failed to quit using nicotine replacement prior to the study; 42% of the sample had used ECs before the study.

In the absence of published direct comparisons between varenicline and ECs, a meta-analysis was not done. This study was approved by the Johns Hopkins University Institutional Review Board and registered on PROSPERO [ID: CRD42020157378].

Results

Our initial literature search generated a total of 461 articles. Of the 461 articles searched, reviewed, and screened, 35 articles were found to be related to our research objectives while 426 articles were excluded. Twenty (22) relevant pieces of information were found from government and non-government agency websites, and 14 data-driven articles met the eligibility criteria for systematic reviews and were eventually included in the final qualitative synthesis (Figure 1). The updated literature search generated an additional three articles, two of which were appropriate for the synthesis; one other paper was identified from the reference list of one of these two papers, and yet another paper from the reference list of the third included paper - so four papers in all were included based on the updated search. Based on the Oxford Quality Scale [30] used to assess the quality of the RCTs reviewed, the overall average quality ratings for the RCTs fall in the medium to high range.

In an RCT conducted by Jorenby et al. on the safety and efficacy of varenicline vs bupropion vs placebo as smoking cessation pharmacotherapy, varenicline was shown to consistently increase the chances of quitting and was also observed to be safer in the short- and long-term compared to bupropion and NRT [17]. These outcomes were also observed in a similar RCT conducted by Gonzales et al. (Table 2) [18]. Another RCT on the safety of smoking cessation medication led by Anthenelli was conducted in 140 centers spanning 16 countries over a three-year study period from November 2011 through January 2015 [25]. The authors evaluated neuropsychiatric safety and the efficacy of varenicline, bupropion, and nicotine patches. Each medication was compared with placebo; the study involved 8,144 participants randomized within a psychiatric and a non-psychiatric cohort. In the non-psychiatric cohort, major neuropsychiatric adverse effects occurred in 1.3% of the varenicline group compared to 2.2%, 2.5%, and 2.4% in the bupropion, nicotine, and placebo groups, respectively. In the psychiatric cohort, which included persons with varied psychiatric diagnoses except for alcohol or substance use disorders, major neuropsychiatric events were detected in 6.5% of the varenicline group versus 6.7%, 5.2%, and 4.9% in the bupropion, nicotine, and placebo groups, respectively. There was no significant increase in neuropsychiatric adverse events attributed to varenicline. With respect to efficacy, compared to placebo, varenicline achieved higher continuous smoking abstinence with an odds ratio (OR) of 3.61 compared to that of bupropion vs placebo, OR 1.75. NRT was the least efficacious (OR 1.68) when compared to placebo over the same period (Table 2) [25]. 

Only a few RCTs on the effectiveness of ECs have been completed to date. The most cited study is the RCT conducted by Christopher Bullen and his colleagues over a two-year period among smokers wanting to quit in New Zealand [22]. The authors randomized 657 subjects into three groups: nicotine ECs, NRT, and placebo ECs. In the nicotine EC group, a higher proportion (7.3%) of participants were verified to have quit smoking at six months compared to 5.8% in the NRT and 4.1% in the placebo ECs group, but the results were not statistically significant. The authors, therefore, concluded that nicotine ECs were modestly as effective as NRT in smokers who are eager to quit (Table 3). In a survey of 216 adult smokers who were not using ECs at the beginning of the study, more than two-thirds (67%) of study participants reported a reduction in the number of cigarettes smoked after initiation of e-cigarettes, and 31% stopped smoking at six months [34]. However, in one meta-analysis of 20 studies that was designed to compare the efficacy of nicotine ECs and NRT among cigarette smokers, with the primary endpoint as smoking cessation, subjects who used ECs had 28% lower odds of quitting smoking compared to those who used NRT [35] (Table 3). Yet, a national survey of 2,254 active adult smokers showed that a significantly higher proportion (83%) of the respondents who used ECs had prior failed quit attempts compared to 74% of non-users (Table 3) [36].

A longitudinal web-based survey involving 374 former smokers followed for 15 months was aimed at assessing the characteristics affecting relapse. Among ex-smokers who used ECs during the 15-month period, non-daily use was associated with higher odds of relapse than daily use [41]. Another cross-sectional study showed a negative relationship between quit success and e-cigarette ever use, but a positive relationship with current e-cigarette use measures [42]. Furthermore, an international cross-sectional survey involving 1,070 participants from Canada, the United States, England, and Australia compared indicators of prior and current cigarette smoking dependence and of relapse in former smokers who were daily ‘vapers’ or who were not vaping at the time of the survey. Compared to former smokers and current non-vapers, former smokers who currently vape nicotine daily reported higher levels of cigarette smoking dependence pre- and post-cessation, as well as greater confidence in staying quit and similar strength of urges to smoke [43]. Finally, a randomized controlled trial of 886 smokers in the United Kingdom who had access to four weeks of weekly one-on-one behavior support showed that 18% in the EC group were biochemically confirmed abstinent at 52 weeks, compared to 9.9% in the nicotine replacement group [44]. In this study, nicotine replacement (type chosen by the participant, with the option to combine two types) was provided to the assigned group for three months; a starter kit of a refillable EC and 30 ml of nicotine-containing e-liquid was provided to EC participants with advice that they buy additional e-liquid when needed. At 52 weeks, 39.5% in the EC group were still using ECs compared to 4.3% in the nicotine replacement group that remained on nicotine replacement [44].

Discussion

We found no head-to-head RCT comparing varenicline to ECs. Both items have data suggesting efficacy for smoking cessation, though this was not statistically significant in all the studies on ECs. A number of questions remain unanswered.

Are Electronic Cigarettes Effective as Smoking Cessation Aids?

Is it possible that the drop in traditional smoking prevalence from 2007 to date is at least partly the result of adoption, effectiveness, or the wide availability of electronic cigarettes? Should (nicotine-containing) electronic cigarettes be considered nicotine replacement therapy? The highest prevalence of active electronic cigarette users in the US in 2018 was among persons aged 18-24 years [3]. Among current smokers in one study, 24% reported e-cigarette use [36]; among current electronic cigarette users, 52% reported current conventional cigarette use while 19% denied prior cigarette use [45]. Smoking cessation, smoking reduction, and reduction in health risks are the most common reasons for the use of electronic cigarettes [36].

Several clinical studies have evaluated the role of electronic cigarettes as quitting aids, but many of them are prospective observational studies. Findings from these studies are conflicting, possibly due to inherent reporting and confounding biases of observational studies [40]. However, historically, clinical trial subjects are different from real-world patients, as there are many factors that could influence real-world effectiveness. In addition, only specific brands of ECs were tested in the clinical trials out of the hundreds of available EC brands. Indeed, among 15 longitudinal real-world studies assessed by Kalkhoran and Glantz, only six showed statistically significant decreases in smoking cessation with the use of ECs [35]. Although there may be a place for ECs in therapy to modify smoking behavior or reduce the number of cigarettes smoked [34,37-38], the effects of the E-cigarette Vaping Acute Lung Injury (EVALI) outbreak of 2019 on the prevalence of EC use among American youth and adults are yet to be understood.

In August 2019, the US Centers for Disease Control & Prevention (CDC) began tracking an outbreak of EVALI [46]. An Illinois survey study that compared 66 EC users who experienced EVALI to 519 EC users who had not had EVALI found that EVALI patients had higher odds of reporting the exclusive use of tetrahydrocannabinol (THC)-containing products (adjusted odds ratio [aOR] = 2.0, 95% confidence interval [CI] = 1.1-3.6); frequent use (more than five times per day) of these products (aOR = 3.1, 95% CI = 1.6-6.0), and obtaining these products from informal sources, such as a dealer, off the street, or from a friend (aOR = 9.2, 95% CI = 2.2-39.4) [47]. The odds of using Dank Vapes, a class of largely counterfeit THC-containing products, were also higher among EVALI patients (aOR = 8.5, 95% CI = 3.8-19.0). An interview and chemical analysis study in Minnesota found that 67% of EVALI patients interviewed had used Dank Vapes, and that Dank Vapes and most other products that were submitted by EVALI patients or seized by law enforcement after raids in 2019 contained vitamin E acetate; bulk liquids and cartridges obtained in a 2018 raid did not contain vitamin E acetate [48]. In a study across 17 states, vitamin E acetate was found in bronchoalveolar lavage samples of all 25 confirmed EVALI cases, 23 of 26 probable EVALI cases, and none of 99 healthy comparators that included 18 EC users [46].

Among 96 patients with confirmed or probable EVALI assessed in Minnesota up till October 31, 2019, the median age was 21; range being 15-71 [48]. In a national analysis of hospitalizations for confirmed or probable EVALI, 2558 nonfatal and 60 fatal cases had been reported by January 7, 2020; the age range among fatal cases was 15-75 [49]. Compared to nonfatal EVALI cases, EVALI fatalities had a higher prevalence of asthma, chronic obstructive pulmonary disease, heart disease, or a mental health condition other than substance use disorder [49].

Efficacy of Varenicline as a Smoking Cessation Intervention

The observed low smoking cessation rates attending all quit attempts have been attributed in part to the intolerable effects of nicotine withdrawal. Varenicline is a selective partial agonist at the α4β2 nicotinic acetylcholine receptor subtype, where it stimulates dopamine secretion to reduce nicotine withdrawal symptoms. It, therefore, helps counteract nicotine withdrawal symptoms by blocking the action of nicotine on the brain [28]. After the FDA placed a black box warning on varenicline in 2009 due to concerns about neuropsychiatric side effects, varenicline sales declined significantly [50]. However, based on the results of the EAGLES trial discussed above [25], the FDA lifted the black box warning in 2016. Subsequently, varenicline prescriptions have risen among Veterans Health Administration and Medicaid patients [51], and by extension, smoking prevalence should continue to fall.

Recommendations

Several of the available studies underscore the need for additional research to unequivocally establish the overall benefits and harms of e-cigarettes at both individual and population levels [22,37,39]. Any policy provisions on e-cigarettes based on the conclusions reached by these studies would therefore be preemptive, and the rigor of these studies as well as the potentially biased nature of their positions (see Table 3) should be the focus of discussion by policymakers who intend to draw policy conclusions from such studies. While respecting the choice of some smokers to use e-cigarettes as an alternative smoking cessation method following the failure of initial cessation treatment attempts, the American Heart Association (AHA) suggested clinicians inform patients of the potentially toxic chemicals in e-cigarettes, that e-cigarettes have not been proven to be effective smoking cessation agents, and that they should consider setting a realistic quit date for using e-cigarettes [19]. The AHA has also advocated for incorporating e-cigarettes in statutory state and federal laws, especially regarding sales to minors, as has the American College of Physicians (ACP) [20].

The FDA has approved several products, as smoking cessation aids to help reduce dependence on nicotine, including varenicline. The FDA recognized, as it announced the rule regulating electronic cigarettes, that they have both potential benefits and risks until future research proves otherwise [13]. However, given the 2019 EVALI outbreak, the health of millions of Americans could be in jeopardy [3,35,40], especially since an estimated 5.3 million U.S. middle and high school students currently used ECs as of 2019 [14].

A key aspect of the debate about how ECs should be regulated has been the concern that widespread use of these devices could lead to the renormalization of smoking and consequently, the regulation of ECs should mirror that of conventional cigarettes [11]. Although ECs are generally seen as exposing users to fewer toxicants than do conventional cigarettes, concern about health risks for users has led the American Diabetes Association to discourage EC use in persons with diabetes [21]. As of April 2020, FDA guidance includes the following:

People should not use THC-containing e-cigarette, or vaping, products, particularly from informal sources like friends, or family, or in-person or online dealers.

Vitamin E acetate should not be added to any e-cigarette, or vaping, products. Additionally, people should not add any other substances not intended by the manufacturer to products, including products purchased through retail establishments.

Adults using nicotine-containing e-cigarette, or vaping, products as an alternative to cigarettes should not go back to smoking; they should weigh all available information and consider using FDA-approved smoking cessation medications. They should contact their healthcare professional if they need help quitting tobacco products, including e-cigarettes, as well as if they have concerns about EVALI.

E-cigarette, or vaping, products should never be used by youths, young adults, or women who are pregnant. Adults who do not currently use tobacco products should not start using e-cigarette, or vaping, products [52].

Rising e-cigarette use among youth and young adults is yet a major public health concern in the U.S. The greatest public health concern about e-cigarettes, however, is not the rate at which youth are currently using e-cigarettes, but the rate at which youth use of e-cigarettes may increase rates of youth use of more harmful tobacco products. The concern about non-smoking youth who use e-cigarettes becoming smokers [53-54] is a valid, data-driven one requiring urgent public health intervention and policy consideration. Within the context of the COVID-19 pandemic, ECs may enhance respiratory disease caused by SARS-CoV-2 due to their potential acute pulmonary toxicity. As such, suspension of the use of e-cigarettes during the period of SARS-CoV-2 circulation or at least from the onset of symptoms has been recommended [55]. Given the current limited understanding of the long-term health effects of ECs therefore, prudent public health policy should aim at reducing the marketing of e-cigarettes to youth.

Although varenicline may not be the perfect panacea for smoking cessation in adults, with only a 44% quit rate at best [17-18,27], it currently appears to be better than e-cigarettes as a smoking cessation agent despite a drop in its prescription rates by about 76% between 2007 and 2014 [50], likely due to the effect of the (now-rescinded) black box warning. What may hinder the uptake of varenicline at the moment as an ideal smoking cessation agent is the recent discovery of the carcinogen N-nitrosodimethylamine (NDMA) in some lots of the medication (Chantix^R^, U.S; Champix^R^, Canada). Although health authorities in Canada have issued a recall on Champix, FDA is yet to officially issue a recall on Chantix. Notwithstanding, Pfizer has decided to suspend its production as an anti-smoking agent until further analysis is conducted [56]. Conversely, in a recent recommendation statement released by The United States Preventive Services Task Force (USPSTF), there is currently insufficient evidence to assess the balance of benefits and harms of e-cigarettes for tobacco cessation in adults and pregnant individuals. Clinicians are therefore encouraged to direct patients who use tobacco to other tobacco cessation interventions with evidence-based effectiveness and safety [57].

With respect to the foregoing, and based on available evidence, we make the following recommendations:

1. A Healthy People objective that seeks reduction of EC use among minors should be established and pursued. Meanwhile, knowledge gaps on E-cigarettes should be bridged by more rigorous government and non-government agency-funded research on the true biochemical components of e-cigarettes, their potential toxicities, and the short- and long-term health effects on users and non-users.

2. There should be appropriate media messaging and warning labels about e-cigarettes, as well as the prohibition of flavors and the banning of sports and entertainment sponsorships.

3. The recent discovery of the carcinogen, N-nitrosodimethylamine (NDMA), in varenicline (Chantix^R^, U.S; Champix^R^, Canada) may hinder the uptake of varenicline at the moment as an ideal smoking cessation agent until full risk mitigation is completed.

4. Given that there is currently insufficient evidence to assess the balance of benefits and harms of e-cigarette for tobacco cessation in adults and pregnant individuals, clinicians should direct patients who use tobacco to other tobacco cessation interventions with evidence-based effectiveness and safety [57].

5. Following the FDA’s removal of varenicline’s black box warning and given the evidence in its support as a first-line smoking cessation agent, we support policy consideration for reclassifying varenicline as over the counter (OTC) as recently discussed by Leischow [58], realizing that a study of safety and efficacy of varenicline as an OTC medication may run till 2022 [59].

Adults are perceived to use e-cigarettes as a smoking cessation aid while youths are thought to use them recreationally. However, the use of ECs has been shown to correlate with increased risk for cigarette initiation and use among youths, and the use of ECs has rapidly expanded despite concerns about safety, dual-use, and possible 'gateway' effects. The American Diabetes Association (ADA) does not consider e-cigarettes to be an alternative to smoking or an aid for smoking cessation, and it advocates that all persons with diabetes should abstain from using tobacco products and e-cigarettes [21]. Therefore, based on currently available evidence and pending additional research, the use and sale of ECs, like other tobacco products, should be monitored and regulated.

Strengths and limitations

This paper is unique as it appears to be the first review to compare varenicline with e-cigarettes. In addition, the overall average quality ratings for the included randomized controlled studies fall in the medium to high range. However, although three meta-analyses were cited [32-33,35], we did not conduct a meta-analysis for this systematic review due to the paucity of relevant methodologically sound studies (i.e., best evidence synthesis) at the time of data extraction. In addition, the current lack of prospective data on the impact of e-cigarettes on acute respiratory infections precludes a full discussion on a potential impact on the severity of acute viral respiratory infections like COVID-19.

## Conclusions

Given the current lack of clear evidence in support of e-cigarettes as an effective smoking cessation aid, clinicians should direct patients who use tobacco to other evidence-based tobacco cessation interventions. Following FDA’s removal of varenicline’s black box warning, a recent statement from the American College of Cardiology supporting it as a first-line smoking cessation agent, and the manufacturer’s plan to address the impurity (NDMA) recently found in Chantix^R^, we anticipate an increase in its prescription and uptake rates over time.
